# Targeted Formation of Biofilms on the Surface of Graphite Electrodes as an Effective Approach to the Development of Biosensors for Early Warning Systems

**DOI:** 10.3390/bios14050239

**Published:** 2024-05-09

**Authors:** Anna Kharkova, Roman Perchikov, Saniyat Kurbanalieva, Kristina Osina, Nadezhda Popova, Andrey Machulin, Olga Kamanina, Evgeniya Saverina, Ivan Saltanov, Sergey Melenkov, Denis Butusov, Vyacheslav Arlyapov

**Affiliations:** 1 Federal State Budgetary Educational Institution of Higher Education, Tula State University, 300012 Tula, Russia; anyuta_zaytseva@mail.ru (A.K.); r.n.perchikov@tsu.tula.ru (R.P.); kristina-syundyukova@yandex.ru (K.O.); basiblab@tsu.tula.ru (O.K.); 2 N. D. Zelinsky Institute of Organic Chemistry, 119991 Moscow, Russia; s.k.kurbanalieva@tsu.tula.ru (S.K.); e.a.saverina@tsu.tula.ru (E.S.); 3 Federal State Budgetary Institution of Science Institute of Physical Chemistry and Electrochemistry of the Russian Academy of Sciences, 119071 Moscow, Russia; nm.popova.ipce.ras@gmail.com; 4 Institute of Biochemistry and Physiology of Microorganisms of the Russian Academy of Sciences—A Separate Subdivision of the FRC Pushchino Scientific Center for Biological Research of the Russian Academy of Sciences, 142290 Pushchino, Russia; and.machul@gmail.com; 5 Limited Liability Company “INNOBIOSYSTEMS”, 117342 Moscow, Russia; sivs@yandex.ru (I.S.); melenkov.s@yandex.ru (S.M.); 6 Computer-Aided Design Department, Saint Petersburg Electrotechnical University “LETI”, 197022 Saint Petersburg, Russia; dnbutusov@etu.ru

**Keywords:** biofilms, *Pseudomonas veronii*, *Saccharomyces cerevisiae*, *Escherichia coli*, BOD biosensors

## Abstract

Biofilms based on bacteria *Pseudomonas veronii* (*Ps. veronii*) and *Escherichia coli* (*E. coli*) and yeast *Saccharomyces cerevisiae* (*S. cerevisiae*) were used for novel biosensor creation for rapid biochemical oxygen demand (BOD) monitoring. Based on the electrochemical measurement results, it was shown that the endogenous mediator in the matrix of *E. coli* and *Ps. veronii* biofilms and ferrocene form a two-mediator system that improves electron transport in the system. Biofilms based on *Ps. veronii* and *E. coli* had a high biotechnological potential for BOD assessment; bioreceptors based on such biofilms had high sensitivity (the lower limits of detectable BOD_5_ concentrations were 0.61 (*Ps. veronii*) and 0.87 (*E. coli*) mg/dm^3^) and high efficiency of analysis (a measurement time 5–10 min). The maximum biosensor response based on bacterial biofilms has been observed in the pH range of 6.6–7.2. The greatest protective effect was found for biofilms based on *E. coli*, which has high long-term stability (151 days for *Ps. veronii* and 163 days for *E. coli*). The results of the BOD_5_ analysis of water samples obtained using the developed biosensors had a high correlation with the results of the standard 5-day method (R^2^ = 0.9820, number of tested samples is 10 for *Ps. veronii*, and R^2^ = 0.9862, number of tested samples is 10 for *E. coli*). Thus, biosensors based on *Ps. veronii* biofilms and *E. coli* biofilms could be a novel analytical system to give early warnings of pollution.

## 1. Introduction

The amount of dissolved oxygen consumed over a set time and under certain conditions during the biochemical oxidation of organic substances contained in water is normalized using the indicator of BOD [1,2]. Depending on the duration of the analysis, there is total BOD (BOD_tot_) and, for a limited period of time, for example, BOD_5_ for 5 days and BOD_20_ for 20 days. BOD_5_ and BOD_20_ indicators are important trophic saprotrophic–saprobic indicators of water quality for fisheries, allowing for the assessment of habitat conditions for aquatic organisms [3]. Standard methods for determining BOD involve measuring the difference in dissolved oxygen concentrations before and after the incubation of a water sample using amperometric or manometric tests [1,2]. It should be noted that oxygen electrodes from various manufacturers and manometers, for example, OxiTop (WTW, Troistedt, Germany) or OxiDirect (Tintomete, Berlin, Germany), do not resolve the main drawback of the technique—the duration of the analysis. The only possible option to fundamentally reduce the analysis time of BOD assays to several minutes is microbial biosensor application [3,4].

The first models of BOD biosensors were based on surface modification of Clark’s amperometric oxygen electrode with suspension microorganisms (first-generation biosensors) [5,6]. As a result of the organic compound oxidation under the action of microorganisms, dissolved oxygen is consumed. When servicing first-generation biosensors, it is necessary to pre-aerate the samples before conducting the experiment, since the response of the biosensor depends on the initial amount of dissolved oxygen in the sample. In addition, the diffusion exchange of oxygen between the sample and the atmosphere during measurements is inevitable. In recent years, devices based on the generation of a biocatalytic current due to the transfer of electrons, which is formed as a result of the oxidation of organic substances on the surface of the transducer, have been used to determine BOD_5_ [7,8]. This technology is widely used in microbial fuel cells (MFCs) [9,10]. The long-term stability of MFCs is one of the attractive advantages of using this technology in biosensor analysis, achieved through stable electroactive biofilms with high oxidative activity; it allows us to measure BOD for an average of 90–100 days [11]. The MFC substrate levels give two different types of output signaling concentrations: abstracted electrons (via the anode) and the production of protons (by the cathode), which allows MFCs to effectively determine the analyte concentration. Response time is one of the challenges of this technology (the average response time is 140–180 min) [11], but the flow mode of MFCs is also an effective solution as a result of the optimization of the flow rate and the anode chamber volume [12,13]. Another way to achieve a rapid response on a small scale can be realized with bioanodes of a typical MFC with a counter Pd electrode and/or silver/silver chloride cell with an applied voltage; BOD is estimated through amperometric analysis (current–time plots) [14,15], but in this approach, planktonic systems are used, which are not as stable as biofilm MFCs.

**Figure 1:**
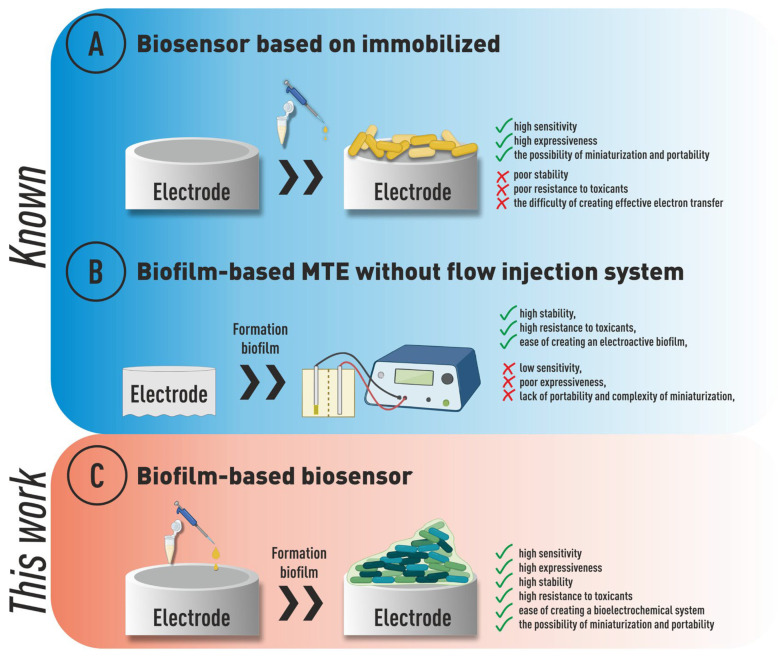
The approach used in this work for the formation of biosensor early warning systems.

This advantage is provided by forming bio-anodes using electroactive or electrochemically active biofilms that are capable of exchanging electrons directly with conductive materials. When forming a biofilm, microorganisms self-produce an extracellular matrix consisting of polymeric substances with electrical activity [16]. Biofilms have been used for BOD analysis in a number of papers published in recent years [17,18,19]. In Ref. [20], a biofilm reactor was developed for BOD determination. The microbial source used for biofilm creation was Weiming pond water. It was shown that adding NaCl to the cultivation allowed for the formation of sensors for rapid BOD determination in seawater. The formed biosensor made it possible to evaluate BOD_5_ in the linear range from 1.0 mgO_2_/dm^3^ to 10 mgO_2_/dm^3^. The system was tested on seven surface and seawater samples; the results of the biosensor assay were roughly similar to those of traditional BOD_5_ results, but the errors of the seawater assessment were greater than those of the freshwater samples. Thus, microbial biofilms represent an excellent alternative to an immobilized suspension of microorganisms for a BOD biosensor receptor system [21,22]. They make it possible to achieve high stability of the biosensor output, avoid the influence of external factors (changes in pH and temperature, and contact with sample components) [16], and provide high long-term stability to the system [23,24]. In addition, multispecies biofilms have a very wide range of oxidizable substrates, which allows for high correlation with the standard BOD_5_ method [17,18,25] (Figure 1).

However, at the moment, the majority of work about biofilm application for rapid analysis of BOD is based on the use of microbial fuel cells [3]. Without a flow-type structural design of MFCs, despite the advantages listed above, such devices also have a number of disadvantages: long analysis time, reaching up to 10 h, low analysis sensitivity, which does not allow us to analyze relatively clean surface waters, and complexity of the design, which does not provide portability and does not allow for analysis “on site” [8,26]. 

Continuous small-scale flow MFCs are very dependent on stability and flow rate; these parameters have significant effects on the sensor response and analytical performances. In [12,13], to provide high long-term stability (more than 60 days) to the flow MFC system, an injected effluent is needed and a lot of wastewater is collected.

In our research, we used batch mode preparation of bioanodes; the time taken to cultivate the biofilm was optimized by using microorganisms with the highest respiratory activity. To decrease the limiting effect of the disconnected biofilm with the electrodes, a mediator was added into the anode construction. We used *Pseudomonas veronii*, *Escherichia coli*, and a yeast, *Saccharomyces cerevisiae*, for BOD assessment and electrochemical studies through a CV test which will help us to understand how to produce even a small quantity of an electroactive compound. The aim of this study is directed toward biofilm formation based on bacteria *Pseudomonas veronii* and *Escherichia coli* and yeast *Saccharomyces cerevisiae* for BOD assessment. In this paper, we consider for the first time the influence of two approaches to the formation of bioreceptor elements based on the characteristics of a BOD biosensor: the classical one based on immobilized microorganisms and the innovative one based on biofilms. We assume that the created bioelectrochemical system will combine all of the advantages of BOD-MFC systems and will have high speed, sensitivity, and portability.

## 2. Materials and Methods

### 2.1. Reagents and Materials

D-glucose (Panreac, Barcelona, Spain), peptone (Condra, Barcelona, Spain), tryptone (Condra, Barcelona, Spain), and yeast extract (Helicon, Moscow, Russia) were applied as nutrients for biofilm cultivation. Graphite powder (Fluka, Berlin, Germany), paraffin oil (Fluka, Berlin, Germany), ferrocene (Dia-m, Moscow, Russia), and a dialysis membrane with a transmission limit of 14 kDa (Roth, Dautphetal, Germany) were used for working graphite paste electrode (GPE) formation. Sodium–potassium phosphate buffer solution, pH = 6.8, was prepared from 33 mM KH_2_PO_4_ and 33 mM Na_2_HPO_4_ (Dia-m, Moscow, Russia).

### 2.2. Microorganisms Used

The bacteria *Pseudomonas veronii* VKM B-3835 (DSM 11331^T^) were isolated from activated sludge, as described in [27]. The yeast *Saccharomyces cerevisiae* VKM Y-1173 was provided by the All-Russian Collection of Microorganisms of the Institute of Biochemical Physics of the Russian Academy of Sciences, and the bacterium *Escherichia coli* K802 was provided by the Biosensor Laboratories of the Institute of Biochemical Physics of the Russian Academy of Sciences.

### 2.3. Cultivation of Microorganism Cells

Cells of *Pseudomonas veronii* VKM B-3835 and *Escherichia coli* K802 strains were grown on LB medium with the following composition: tryptone—10 g/dm^3^, yeast extract—5 g/dm^3^, and sodium chloride—10 g/dm^3^. The yeast *Saccharomyces cerevisiae* VKM Y-1173 was grown on a rich mineral medium (liquid glucose–peptone nutrient medium). The composition of the liquid medium was as follows: glucose—6.25 g/dm^3^, peptone—6.25 g/dm^3^, yeast extract—3.75 g/dm^3^, and K_2_HPO_4_—0.35 g/dm^3^. The medium was autoclaved at 1.15 atm for 45 min.

Cells were cultivated at 29 °C for 20–24 h. Using an Eppendorf centrifuge (Hamburg, Germany), the biomass was separated at 10,000 rpm (10 min) and then it was washed twice with phosphate buffer, pH = 6.8. The washed biomass was stored at –10 °C in micro test tubes.

### 2.4. Working Mediator Electrode Formation

A plastic tube with a surface area of 6.3 mm^2^ was used to form a working GPE. Modified graphite paste with the following composition, 90 mg of graphite powder, acetone solution of ferrocene at 10 mg/500 μL, and 10 mL of mineral oil, was used for filling the tube.

Immobilized microorganisms at 330 mg/mL in an amount of 10 μL were added onto the electrode surface and fixed by a dialysis membrane. 

To form biofilms on the electrode surface, the electrode was put into sterile tubes with 15 mL of LB nutrient medium for *Escherichia coli* and *Pseudomonas veronii* cultures and peptone–yeast medium for *Saccharomyces cerevisiae*. Biofilm cultivation was carried out at 29 °C and then the formed biofilm was fixed by a dialysis membrane.

### 2.5. Biosensor Measurements

**Figure 2:**
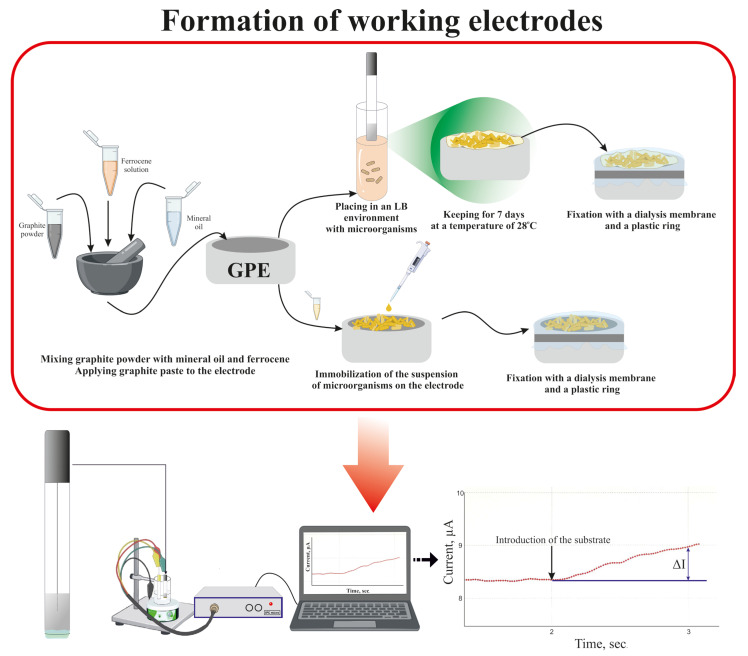
The formation of working electrodes based on a suspension of microorganisms and in the form of a biofilm.

An IPC micro galvano/potentiostat (NTF Volta, St. Petersburg, Russia) was used for electrochemical measurements. The applied potential was +250 mV for ferrocene electrodes. The measurements were carried out at 20 °C with stirring at 300 rpm. After a stable current level was obvious, the test sample was added into the cell. After the amplitude current was recorded, the cell was washed with buffer solution, pH = 6.8 (Figure 2).

### 2.6. Voltametric Measurements

Ecotest-VA (Ltd. Econiks-Expert, Moscow, Russia) was applied to record voltammograms (CVs) with the tested working electrode, a platinum counter electrode, and a saturated silver chloride reference electrode. The measurements were produced at a scan rate of 20–100 mV/s in buffer solution, pH = 6.8.

### 2.7. Scanning Electron Microscopy (SEM)

A JSM-6510 LV scanning electron microscope (JEOL, Tokyo, Japan) was used for the electron microscopic analysis of the samples in a low vacuum (30 Pa) mode of secondary electron registration.

### 2.8. Energy Dispersive X-ray Spectroscopy (EDX)

Element mapping was performed using the Bruker EDS set-top box on a Hitachi TM 4000 microscope (Bruker, Karlsruhe, German), with an accumulation time of 5 min and an accelerating voltage of 15 kV.

### 2.9. Optical Microscopy

A Nikon Eclipse Ci optical light microscope (Nikon, Tokyo, Japan) equipped with a ProgRes SpeedXT core5 camera (Jenoptik, Jena, Germany) was used to examine biofilm formation in the phase contrast mode.

### 2.10. Laser Confocal Scanning Microscopy (LCSM)

A Leica SP5 microscope (Leica, Munchen, Germany) was used for biofilm visualization. Alexa Fluor™ 488 conjugate of Con A (W11261 ThermoFisher, Waltham, MA, USA) [28] was applied to stain the polysaccharide matrix. SYTO^®^ 11 fluorescent dye (S7573 ThermoFisher, USA) was diluted in phosphate buffer at a ratio of 1:1000 and was used for cell visualization. The Nomarski contrast method was applied to record the images: for polysaccharide matrix detection, an argon laser was put at 488 nm, and for cell visualization, the laser was put at 594 nm.

### 2.11. Respiratory Activity of Microorganisms upon Biofilm Growth

The respiratory MTT assay [29] was used for the analysis of the metabolic state of microorganisms in the biofilm. The biofilm was grown in sterile tubes at 29 °C. To control the formation of the biofilm, 0.5 mL of a 0.1% tetrazolium dye 3-(4,5-dimethylthiazol-2-yl)-2,5-diphenyl-tetrazolium bromide (MTT) solution was added and incubated at 29 °C for 1 h. Then, the liquid layer was removed, the biofilm was washed with water, and then ethanol (96%) was added for dye extraction for 45 min. An Expert-003 photometer (Ltd. Econiks-Expert, Russia) was used for the extraction of optical density measurements at 590 nm.

### 2.12. Determination of BOD by the Standard Dilution Method

BOD_5_ of nature samples was measured by using standard protocols [1,2]. An EXPERT-001-4.0.1 BOD thermooximeter (Ekoniks-expert Ltd., Russia) was used to measure the dissolved oxygen content.

## 3. Results

### 3.1. Biofilm Formation

**Figure 3:**
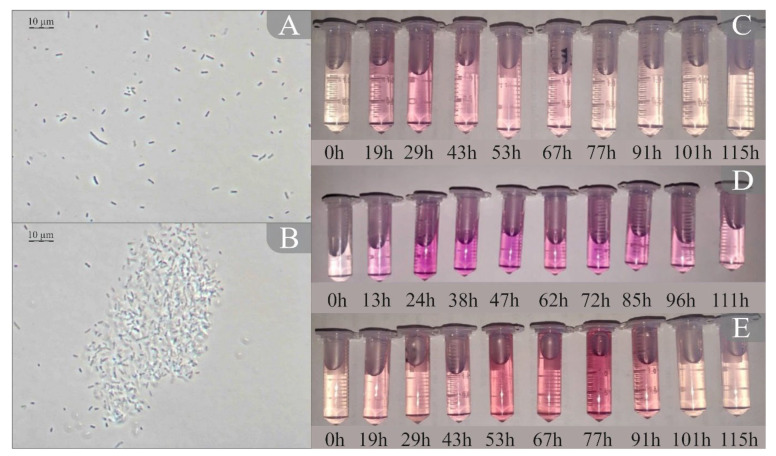
Microscopic studies of the formation of a biofilm of *P. veronii* DSM 11331^T^: (**A**) a photograph of the optical microscopy for immobilized microorganisms of; (**B**) a photograph of the optical microscopy for the biofilm after 47 h of growth. The results of the respiratory MTT assay during biofilm formation: (**C**) *E. coli*, (**D**) *P. veronii*, and (**E**) *S. cerevisiae*.

In this work, the bacteria *Pseudomonas veronii* VKM B-3835, the yeast *Saccharomyces cerevisiae* VKM Y-1173, and the bacterium *Escherichia coli* K-802 were used as a base for a BOD biosensor because they form ecologically and clinically significant biofilms and are the standard for studying biofilms [30,31,32]. They metabolize different substrates and therefore are widely used for BOD analysis [27,33,34]. In an in vitro experiment, the growing of biofilms on the sensitive element surface was controlled by microorganism physiological activity, which was determined by using the respiratory MTT assay. Biofilm growth was assessed by optical density rising, and the optical density dependence on growth time was plotted (Figure 3).

As one can see from Figure 3B, the *Pseudomonas veronii* biofilm after 47 h of growth has a clearly visible polysaccharide matrix, and boundary line between the biofilm and free-living microorganisms is also observable. The morphology of the bacteria in the biofilm is the same as that of the immobilized microorganisms.

The process of increasing the physiological activity is indicated by the increase in optical density, which occurs due to the formation and growth of a biofilm. The time taken to reach the maximum physiological activity of the used microorganisms was 47 h for *Ps. veronii*, 29 h for *E. coli*, and 77 h for *S. cerevisiae*. After these periods of time, a gradual decrease in activity could be observed. Optical microscopy with phase contrast confirmed the formation of biofilms during the period of maximum physiological cell activity (Figure 3C,D).

**Figure 4:**
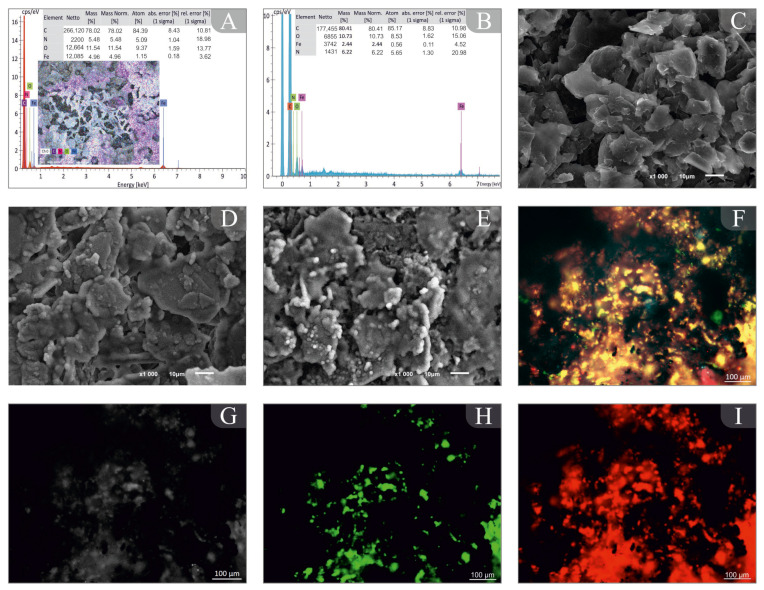
Microscopic studies of the formation of biofilms: (**A**) elemental distribution of the biofilm made from *P. veronii* DSM 11331^T^ at the GPE; (**B**) elemental distribution of a pure GPE; (**C**) the surface of a clean GPE, obtained by SEM; (**D**) *E. coli* biofilm, obtained by SEM; (**E**) *P. veronii* biofilm, obtained by SEM; (**F**) an integral image of the electrode surface for the *P. veronii* biofilm, obtained by the LCSM method; (**G**) an image of the electrode surface in the Nomarski contrast mode for the *P. veronii* biofilm, obtained by the LCSM method; (**H**) an image of the electrode surface in the SYTO 11 dye display mode mode for the *P. veronii* biofilm, obtained by the LCSM method; (**I**) an image of the electrode surface in conA dye display mode for the *P. veronii* biofilm, obtained by the LCSM method.

Figure 4A,B show the elemental distribution of an empty GPE and an electrode with a biofilm; it can be seen that the modification of the electrode occurs successfully since when the film is formed, Fe becomes smaller.

Scanning electron microscopy (Figure 4C–E) was used to confirm biofilm formation on the electrode surface. The biofilm is kept on the GPE surface (Figure 4C) and has a porous structure (Figure 4D,E). The electrode surface is covered by biofilms with a high density and a strong matrix.

Laser confocal microscopy was used to study extracellular polysaccharide matrix formation (Figure 4F–I), which facilitates cell adhesion on the surface of graphite materials. In Figure 4, polysaccharides are red, while nucleic acids are green. Cells on the graphite surface are evenly distributed due to the presence of nucleic acids (Figure 4H). An intense staining of extracellular polysaccharides (Figure 4I) indicates that microorganism set on the electrode surface. The surface of GPEs has been found to have uniform and metabolically active biofilms which can be used as the basis for sensors. 

### 3.2. Study of the Interaction of Microorganisms in Biofilms with an Electron Transport Mediator by Using Cyclic Voltammetry

Investigating the biocatalytic properties of microorganisms during the formation of biofilms is an interesting topic to elucidate their use in biosensor devices. The electron transfer with ferrocene is not pH-dependent, and thus, it was chosen as the mediator in this study. Using the optimal pH for high oxidative activity of different microorganisms is possible with this method. Modifying the graphite paste can be achieved through mediator immobilization on the electrode surface due to ferrocene’s low solubility in water [35,36].

The efficiency of the microorganism interaction with ferrocene was estimated by a rate constant. Cyclic voltammetry and the Nicholson–Shine math model [37] was applied to obtain the rate constant according to Equation (1).

(1) $$\frac{I_{k}}{I_{d}}=\sqrt{\frac{k_{\mathrm{int}}\left[E\right]RT}{nFv}}$$

*I_k_* is the limiting current after adding glucose; *I_d_* is the limiting current in the absence of glucose; *k*_int_ is the rate constant; [*E*] is the cell concentration, mg/L; *R* is the universal gas constant, J/(mol K); *T* is the temperature, K; *n* is the number of transferred electrons; *F* is the Faraday constant, C/mol; *ν* is the scan rate, V/s.

**Figure 5:**
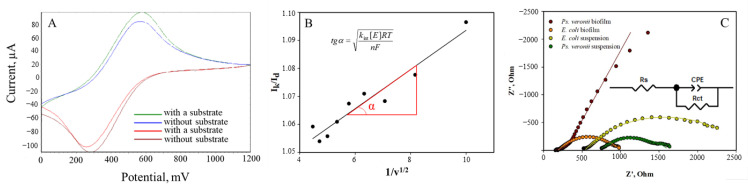
Determination of the interaction constant of the ferrocene mediator with microorganisms in a biofilm through cyclic voltammetry. (**A**) A voltammogram for the *S. cerevisiae* biofilm; (**B**) the dependence of the ratio of limiting currents in the presence and in the absence of a substrate on the reciprocal of the root of the scan rate 1/*ν*^1/2^ for calculating the rate constant of the interaction between the *S. cerevisiae* biofilm and the ferrocene mediator; (**C**) electrochemical impedance spectroscopy for the suspension and the biofilms made from *Ps. Veronii* and *E. coli* at 0.25 V compared to Ag/AgCl. The lines show equivalent circuit fitting.

The plots (Figure 5) of the anode current ratio (*I_k_*/*I_d_*) vs. the values 1/*ν*^1/2^ are used for rate constant determination based on the linear regression slope.

The obtained values of the interaction rate constants in the “biomaterial-mediator-electrode” system are presented in Table 1. Table 1 shows that bacterial biofilms have greater rate constants for interaction with ferrocene compared to immobilized cells.

The *E. coli* biofilm- and the *S. cerevisiae* yeast immobilized microorganism-based receptor systems have the highest rate constant of interaction with the biomaterial. Bacterial biofilms have a greater rate interaction constant between the ferrocene mediator and the microorganisms compared to immobilized cells. Significantly improved contact between bacterial enzymes and the electrode surface can be attributed to this fact. On the other hand, a significant decrease in the rate constant was observed for the *S. cerevisiae* yeast biofilm compared to immobilized yeast.

The equivalent electrical circuits used to fit the spectra are shown in Figure 5C. Pure graphite electrodes and modified electrodes, as they are less complex in architecture, have been successfully described by a circuit containing Rs (electrolyte resistance) connected in series with a parallel combination of R and CPE, where CPE is a constant phase element representing the double layer capacitance of CPE and charge transfer resistance Rct. The constant phase element is modeled as a non-ideal capacitor and is described as CPE = 1/T − (iωC) − α, where ω is the angular frequency, α is the CPE indicator reflecting the inhomogeneous surface, and C is the capacitance. As expected, with the formation of a biofilm on a GPE, the resistance decreases; however, in the biofilm of *Ps. veronii,* resistance increases, which may indicate a greater release of the matrix in which electron transport shuttles are pointwise distributed, which complicates electron transfer.

**Figure 6:**
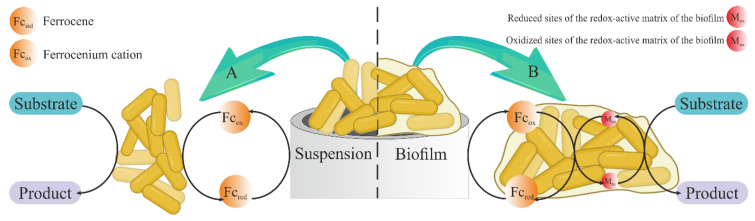
The mechanism of electron transfer in the studied biosensor systems. A—in the system “GPE—ferrocene—immobilized microorganisms”; B—in the “GPE—ferrocene—biofilm of microorganisms” system.

The endogenous mediator of the biofilm matrix and the ferrocene mediator form a two-mediator system that improves electron transport [26,39]. The ferrocene donates electrons to the endogenous biofilm mediator, which is more effective in interacting with microorganism enzyme systems compared to ferrocene (Figure 6). Two-mediator systems have previously demonstrated greater efficiency in bioelectrocatalysis [40]. The lack of electroactive molecules in the matrix that they created that might interact with ferrocene is most likely the cause of the drop in the mediator interaction rate constant in the system based on the *S. cerevisiae* yeast biofilm. The dense matrix surrounding microorganisms makes it difficult for ferrocene to interact directly with cellular enzymes.

### 3.3. Analytical Characteristics of the Developed Bioelectrochemical Systems

**Figure 7:**
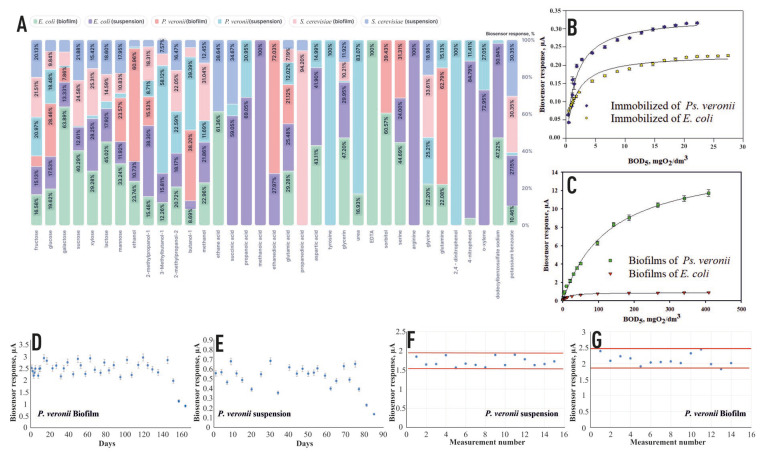
(**A**) A comparative diagram of the substrate specificity of receptor elements based on biofilms and activated sludge; (**B**,**C**) calibration dependencies of the sensor response on BOD_5_ for receptor systems based on immobilized microorganisms and biofilms of the studied microorganisms; (**D**) the long-term stability of *P.veronii* for biofilms; (**E**) the long-term stability of *P.veronii* for suspension; (**F**) the operational stability of *P.veronii* for suspension; (**G**) the operational stability of *P.veronii* for biofilms.

To perform BOD_5_ analysis, it is essential that the created biosensor is able to enlist the biochemical oxidation of a wide range of natural substrates. In this manner, the substrate specificity of microorganisms in the formed conducting frameworks was examined. The results obtained are displayed in Figure 7.

The profile of substrates oxidized by the microorganisms changes during biofilm formation. This effect is particularly pronounced for the two bacterial microorganisms used, *Ps. veronii* and *E. coli*. This effect is also consistent with the model proposed in Figure 5. In the biofilm, the electron acceptors from the active sites of the cellular enzymes are redox compounds released by the biofilm and not ferrocene. In general, it should be noted that the biofilm-based receptor system of *Ps. veronii* has the widest range of oxidizable substrates and is promising for the BOD biosensors’ creation. The yeast *S. cerevisiae* was not used to create a BOD biosensor because of its narrower spectrum of oxidizable substrates. For real-sample analysis, hyperbolic dependences of the sensor response on BOD_5_ were plotted (Figure 7B,C). 

The stability of the sensor response was estimated based on the long-term stability and operational stability. A glucose–glutamic acid solution was selected as the model substrate, which is an internationally recognized reference solution in the measurement of BOD_5_ [1,2]. Table 2 shows the characteristics of the developed mediator biosensors and their analogues.

According to the table, it can be concluded that the BOD biosensors based on *Ps. veronii* and *E. coli* have the best potential for BOD assessment. The developed biofilm biosensors have a stable signal with a relative standard deviation of no more than 10% and a stable operating time of more than 150 days, indicating long-term stability. It should be noted that these systems could be used for natural water assays with low BOD_5_ values, ~1 mg/dm^3^ (pure natural water). The developed biosensors are superior to already known similar products in terms of low detected limits of BOD_5_ [14,18] and long-term stability [42,43], which make them promising for BOD_5_ analysis in surface water and wastewater because of their sensitivity and short test time.

### 3.4. Influence of Negative Environmental Factors on Created Receptor System

In addition to organic molecules that microbes oxidize as part of the bioreceptor system, industrial plant wastewater may also contain inorganic compounds that inhibit the oxidative activity of microorganisms. Therefore, it is crucial to investigate how adverse environmental conditions may affect the operation of any biosensor being developed to measure BOD. The pH of the medium is one factor that affects the activity of cellular enzymes and the sensitivity of the bioreceptor to various substrates. This study investigated variations in the oxidative activity of free-living microorganisms and biofilms at pH values ranging from 5.6 to 7.8. The maximum biosensor response based on bacterial biofilms has been observed in the pH range of 6.6–7.2, which is in accordance with the literature data [27,44]. Microorganisms in biofilms have the most physiological activity in this pH range compared to those in immobilized microorganisms.

**Figure 8:**
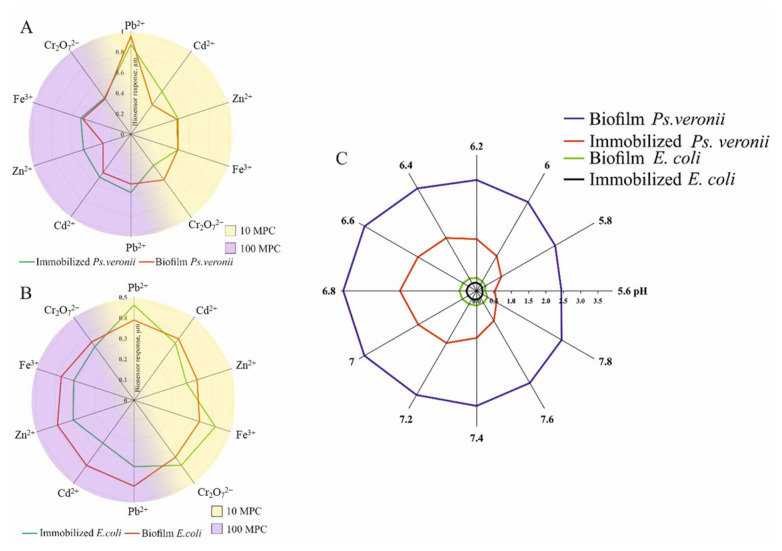
The dependence of the biosensor response based on free-living microorganisms and bacterial biofilms (**A**,**B**) on the presence of heavy metals; (**C**) biosensor response to the pH of the medium.

Heavy metal ions in the wastewater sample could reduce the biosensor response until a complete loss of bioreceptor activity is reached. There are different mechanisms of heavy metal ion inhibition, including binding with functional sulfhydryl groups, physiologically important cation substitutions, etc. [45,46]. The effects of heavy metals on biosensor responses to current glucose–glutamic acid mixtures were examined, and changes in biosensor responses after the addition of Cd^2+^, Pb^2+^, Zn^2+^, Fe^3+^, and Cr_2_O_7_^2−^ were discussed in the concentration range of 10-fold and over 100-fold MPCs in fishery reservoirs [47] (Figure 8).

It can be seen from the presented data that the *E. coli* bacterium, resistant to heavy metals, slightly increases during biofilm formation, which corresponds to the literature data on the resistance of bacterial biofilms [16]. A similar protective effect for *Ps. veronii* is not observed, which is probably because they are resistant to the concentrations of heavy metals used in this study.

### 3.5. Approbation of the Developed Biosensors

**Figure 9:**
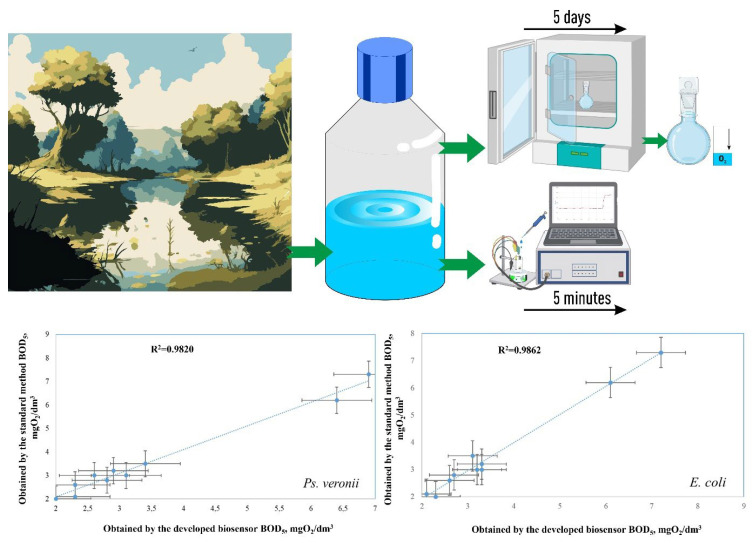
The linear dependence of the results of the BOD_5_ analysis obtained by using the standard method and the developed biosensors based on biofilms.

Ten samples of freshwater and wastewater were used for biosensor approbation. The standard procedure was used to collect samples and determine BOD_5_ in compliance with the most recent regulatory documents (Figure 9).

Modified Student’s test was used for statistical analysis of the obtained data: there were no statistically significant differences between the data of the two different methods. The created biosensors can be applied as prototypes of BOD_5_ monitoring devices.

## 4. Conclusions

This study proposes the use of biofilms based on three microorganisms, bacteria *Ps. veronii* and *E. coli*, and yeast *S. cerevisiae,* to form the receptor element of the BOD biosensor. The methods of SEM, optical microscopy with phase contrast, and laser confocal microscopy proved the formation and fixation of a biofilm on the surface of the GPE. Based on the narrow substrate specificity, biofilms based on *S. cerevisiae* are not very suitable for the determination of BOD. Biofilms based on *Ps. veronii* and *E. coli* for the assessment of BOD have a high biotechnological potential. Bioreceptors based on them are highly sensitive (the lower limit of determined BOD_5_ concentrations is 0.61 and 0.87 mg/dm^3^ for receptor elements based on *Ps. veronii* and *E. coli*, respectively), have a high efficiency of analysis (a measurement time of 5–10 min), and have high long-term stability (151 and 163 days for *Ps. veronii* and *E. coli*, respectively). It has been established that using biofilms enables the creation of bioreceptors with superior analytical capabilities compared to analogues created using the same immobilized microorganisms (classical approach). It was shown that the bioreceptor based on the biofilm of the *E. coli* bacterium was more resistant to heavy metal ions than the similar bioreceptor based on the immobilized microorganisms. The high correlation between the results of the standard analysis of BOD_5_ and those obtained by using biosensors based on the *Ps. veronii* biofilm (R^2^ = 0.9820, n = 10) and *E. coli* biofilm (R^2^ = 0.9862, n = 10), as well as the high analytical capabilities of the receptor systems in the long term, allow for their use as prototypes of BOD_5_ monitoring devices for serial production.

## Figures and Tables

**Table 1:** Rate of interaction constants of microorganisms with ferrocene and data obtained by analyzing impedance spectra of immobilized microorganisms and microorganisms in biofilm at 0.25 V compared to Ag/AgCl using equivalent connection scheme.

Microorganisms	Method of Application to Electrode	Interaction Constant of Ferrocene Mediator with Microorganisms, dm^3^/(g·c)	C, μF	A	Rct, Ohm
*P. veronii*	Biofilm	0.019 ± 0.005	50 ± 2	0.673 ± 0.008	2140 ± 40
	Immobilized cells	0.00020 ± 0.00002 [38]	69 ± 4	0.58 ± 0.01	890 ± 10
*E. coli*	Biofilm	0.14 ± 0.06	40 ± 1	0.66 ± 0.005	806 ± 5
	Immobilized cells	0.030 ± 0.006 [38]	17.8 ± 0.9	0.66 ± 0.008	2080 ± 40
*S. cerevisiae*	Biofilm	0.013 ± 0.004	-	-	-
	Immobilized cells	0.3 ± 0.1 [38]	-	-	-

**Table 2:** Comparison of analytical and metrological characteristics of mediator biosensors.

Biomaterial/Conduction System	Long-Term Stability, Days	Relative Standard Deviation, % (n = 15)	Linear BOD_5_ Range, mg/dm^3^	Analysis Time, min	Reference
Immobilized *Ps. veronii*/ferrocene	77	6.88	1.56–1.7	5–10	This study
Biofilms *Ps. veronii*/ferrocene	151	8.53	0.61–130	5–10	This study
Immobilized *E. coli*/ferrocene	83	6.91	1.39–1.59	5–10	This study
Biofilms *E. coli*/ferrocene	163	7.69	0.87–14	5–10	This study
Biofilm based on a community of microorganisms	-	-	49–723	-	[41]
Electroactive activated sludge biofilm/CNT	53	5.96	0.41–23	5	[42]
Biofilm community	-	-	20–500	-	[42]
*S. cerevisiae* suspension/potassium ferricyanide + vitamin K3	-	4.16	20–225	20	[14]
Bacteria isolated from activated sludge/ferrocene redox polymer/CNT	50	5.0	0.1–4.5	5	[26]

## Data Availability

Data are contained within the article.
